# Prevalence of Extended-Spectrum Beta-Lactamases-Producing Microorganisms in Patients Admitted at KRRH, Southwestern Uganda

**DOI:** 10.1155/2017/3183076

**Published:** 2017-02-08

**Authors:** Baguma Andrew, Atek Kagirita, Joel Bazira

**Affiliations:** ^1^Microbiology Laboratory, Kabale Regional Referral Hospital, P.O. Box 7, Kabale, Uganda; ^2^Department of Microbiology, Faculty of Medicine, Mbarara University of Science and Technology, P.O. Box 1410, Mbarara, Uganda; ^3^Bacteriology Reference Laboratory, Central Public Health Laboratories, P.O. Box 7272, Kampala, Uganda

## Abstract

The emergence of extended-spectrum beta-lactamase- (ESBL-) producing pathogenic bacteria at Kabale Regional Referral Hospital (KRRH), located in southwestern Uganda, is of great concern: a phenomenon that worries clinicians and other healthcare workers due to the serious threat they pose to patients. This current study aimed at determining the phenotypic detection of ESBL-producing strains of* E. coli, Klebsiella* sp., and* Proteus* sp. isolated from clinical specimens and their prevalence in patients admitted at KRRH. We used combined disc diffusion technique to detect and establish the presence of ESBLs-producing bacteria. Of the 100 tested bacterial isolates, 89 (89%) were identified as ESBL-producing bacteria.* Klebsiella *sp. predominated in the samples (46 (52%)), presenting the highest frequency of ESBLs producing followed by* E. coli* (39 (44%)) and* Proteus mirabilis* (4 (4.5%)) from the combined disk diffusion.

## 1. Introduction

Antibiotic resistance of bacteria is commonly seen in daily medical practice with multidrug-resistant Gram-negative bacteria posing the greatest threat to human health [1]. *β*-Lactam antibiotics are the commonly prescribed antibiotics to treat bacterial infections, especially at KRRH. However, most Gram-negative bacteria produce *β*-lactamases enzymes which are their major defense mechanism against *β*-lactam antibiotics [2]. Extended-spectrum *β*-lactamases (ESBLs) are the type of *β*-lactamase enzymes produced by certain Gram-negative bacteria in the family of Enterobacteriaceae [3]. ESBL enzymes give the bacteria ability to resist penicillins and cephalosporins of the first, second, and third generations as well as aztreonam through hydrolysis of these antibiotics [4, 5] and are encoded by mobile genetic elements [6]. Alarmingly, these genes code resistance to not only cephalosporins and penicillin but also other antibiotics such as aminoglycosides, fluoroquinolones, tetracyclines, chloramphenicol, and sulfamethoxazole/trimethoprim [6].

ESBL-producing members of Enterobacteriaceae have gained attention in hospital settings because of the limited therapeutic options to treat them resulting in poor clinical outcome and their increasing capacity to cause hospital acquired infections [7]. Hospitalized patients easily acquire these bacteria and eventually act as reservoir [8].* E. coli, K. pneumoniae*, and* Proteus *sp. are the most common ESBL-producing bacterial species, although other bacterial species in the families of Enterobacteriaceae and Pseudomonadaceae are also known to produce such enzymes [9]. Patients infected with ESBL-producing bacteria are likely not treated with beta-lactam antibiotics owing to the risks of treatment failure leading to death and amplified infectiousness [2, 10]. Therefore, early detection of these bacteria is important to control and prevent nosocomial outbreaks in hospital settings.

The prevalence of ESBLs-producing bacteria is unknown in this region; therefore, this current study aimed at evaluating the prevalence of ESBL-producing bacteria at KRRH, using a phenotypic detection procedure based on the combined disk diffusion method. This method is the cheapest strategy to meet the local demands in resource constrained settings compared to more expensive and unaffordable genotypic detection techniques [11–13]. In addition to that, no data exist about the level of ESBL-producers of certain members of the Enterobacteriaceae family. Therefore, this study also will isolate, detect, and characterize ESBL-producing Enterobacteriaceae bacteria from clinical samples of patients admitted at KRRH. This will offer evidence on the reality of ESBL prevalence in the Ugandan hospitals environment and will represent valuable help to control this emerging problem.

## 2. Materials and Methods

A total of 150 clinical specimens were collected in this cross-sectional study, conducted in one year (May 2015 and June 2016) involving 70 wound swabs, 67 urinary tract infections, and 13 CSF aspirates from female and male patients who were admitted in various wards at Kabale Regional Referral Hospital (KRRH). Sterile dry cotton swabs, universal bottles, and plain vacutainer tubes were used to collect pus, urine, and CSF, respectively, from patients. Urine and CSF specimens were collected aseptically into their respective sterile containers using standard techniques [14]. These specimens were then transported immediately to the laboratory where they were directly inoculated onto MacConkey agar (BD), 5% sheep blood agar, and chocolate agar. The inoculated MacConkey agar (BDH) and 5% sheep blood agar were incubated aerobically at 35–37°C for 18–72 hours, while chocolate agar was incubated in anaerobic condition with similar temperature and time range. The bacterial growth was noted based on colonial characteristics [15, 16] and identified by Gram staining, TSI, and analytical profile index (API20E) [17]. A total of 100 consecutive nonrepetitive bacteria were isolated and identified and these included* K. pneumonia*,* E. coli*,* K. oxytoca*, and* P. mirabilis*. These isolates were further tested for antimicrobial susceptibility and screened for ESBL production followed by double-disk diffusion method to confirm ESBL production using ceftazidime (30 *μ*g) and cefotaxime (30 *μ*g) antimicrobial disks with or without clavulanic acid (10 *μ*g). A zone difference of >5 mm between disks was considered indicative of ESBL production [16].

The isolates were subjected to cefpodoxime (10 *μ*g), ceftazidime (30 *μ*g), and cefotaxime (30 *μ*g) antibiotic disks alone and in combination with clavulanic acid (10 *μ*g) (Oxoid; Basingstoke, UK) to determine the presence of ESBLs by the disc diffusion method on Müller-Hinton agar plates, using respective bacterial suspensions with the turbidity adjusted to a 0.5 McFarland standard. Plates were also incubated at 35–37°C for 18–24 hours.

Results were interpreted according to the guidelines of Clinical and Laboratory Standards Institute (CLSI), where interpretive criteria for ESBL activity were based on an increase of more than 5 mm in the diameter of the inhibition zone around disks containing clavulanic acid as compared to the diameters of the inhibition zone around disks free of this beta-lactamase inhibitor [18]. The zones of inhibition were read and measured using a meter ruler in mm and later recorded into the data collection form. The generated data was analyzed using SPSS 21.0 (Armonk, NY: IBM Corp.) to give descriptive statistics which included frequencies, percentiles, and ratios.

*Klebsiella pneumoniae* ATCC 700603,* Escherichia coli* ATCC 25922, and* Proteus mirabilis* ATCC BAA856 were procured from the Uganda National Health Laboratories (UNHLs) formerly known as Central Public Health Laboratories (CPHL) and used as controls in this study.

### 2.1. Ethical Considerations

Ethical approval was obtained from the research and ethics committee of KRRH. Informed consent was also sought and obtained from the patients. All results were treated with utmost confidentiality.

## 3. Results

A total of 150 clinical samples were tested for culture and antimicrobial sensitivity. Out of the 150 samples, 67 were urine samples, 70 were pus swabs, and 13 were cerebral spinal fluids (CSF). In addition, among the 150 samples, 100 (967%) were identified as growth of Enterobacteriaceae microorganisms. Out of the 100 identified Enterobacteriaceae bacteria, 44 (44%) were detected as* Escherichia coli*, 36 (36%) were* K. pneumoniae*, 15 (15%) were* K. oxytoca*, and the remaining 5 (5%) were* Proteus mirabilis* (Table 1).* E. coli* was the most predominating bacterial pathogen isolated from urine (29 (43%)) and cerebral spinal fluid aspirate (2 (81%)). However,* K. pneumoniae* was the leading bacterial pathogen isolated from the pus specimens (21 (30%)).

The most frequent Gram-negative organisms isolated were* E. coli* (44 (44%)),* K. pneumoniae* (36 (36%)),* K. oxytoca* (15 (15%)), and* P. mirabilis* (5 (5%)).* E. coli* was the most frequently isolated Gram-negative bacteria in all clinical specimens followed by* K. pneumoniae*, whereas* Proteus mirabilis* was the least isolated. Urine specimens had the greatest Gram-negative bacterial growth (53, 79%), while CSF specimens had the least growth of Gram-negative bacteria (3, 23%). However, pus specimens had an amount of only 44 (63%) of Gram-negative Enterobacteriaceae bacterial growth. Among the urine specimens (*n* = 67),* E. coli* (29, 43.3%) was the most frequently isolated Gram-negative bacterial pathogen followed by* K. pneumoniae* (15, 22.4%) and* K. oxytoca* (6, 9.0%), while* P. mirabilis* (3, 4.5%) was the least isolated Gram-negative bacterial pathogen.

In pus specimens (*n* = 70) cultured,* K. pneumoniae* (21, 30%) was frequently isolated followed by* E. coli* (13, 18.6%) and* K. oxytoca* (9, 12.9%), while* P. mirabilis* (1, 1.43%) was the least isolated Gram-negative bacteria. CSF (*n* = 13) was the least among clinical specimens and* Escherichia coli* (2, 15.4%) and* P. mirabilis* (1, 7.69%) were the only Gram-negative bacterial pathogens isolated.

For phenotypic confirmation of potential ESBL-producing bacterial isolates, both cefotaxime and ceftazidime alone and in combination with clavulanic acid by disk diffusion method were used, where a >5 mm increase in a zone diameter for antimicrobial agent tested in combination with clavulanic acid versus its zone when tested alone confirmed an ESBL-producing organism. All the isolated 100 bacterial pathogens were tested. In order to improve the sensitivity, we used ceftazidime, cefpodoxime, cefotaxime, and ceftriaxone antibiotics to screen for ESBL activity. The combined disk test indicated that 89 (89%) isolates presented inhibition of clavulanic acid. The most common species presenting this activity were* K. pneumonia* (33 (92%)),* E. coli* (39 (87%)), and* K. oxytoca* (13 (87%)), while* P*.* mirabilis* was only 4 (80%). Comparing the results of the combinations of cephalosporins with clavulanic acid, those involving cefotaxime (94 (94%)), cefpodoxime (91 (91%)), and ceftriaxone (91 (91%)) were superior to those obtained from when ceftazidime was combined with clavulanic acid (79, 79%) (Table 2).

## 4. Discussion

In a resourced constrained setting like KRRH, the use of combined disk diffusion technique for rapid phenotypic detection and identification of ESBL-producing bacteria is cheapest strategy to meet local demands [19] compared to more expensive and unaffordable genotypic detection techniques. This disk diffusion technique requires only necessary antibiotic discs and appropriate conventional culture media that are used routinely with other routine bacteriology works [20, 21]. Our evaluation showed that the combined disk diffusion technique had a turnaround time of only 48 hours from the time of specimen culture to the final ESBL results, which is an advantage compared to other techniques [22].

Strains of Enterobacteriaceae ESBL-producing enzymes have become a concern in the treatment of infections and infection control programs in hospital settings. Most* K. pneumoniae*,* Klebsiella oxytoca*, and* E. coli* are resistant to ampicillin because of the production of plasmid mediated *β*-lactamase enzymes [15]. Most isolates are susceptible to later generation cephalosporins and aztreonam [5, 6]; however, spontaneous mutations occurrence may result in novel *β*-lactamases which can inactivate extended-spectrum cephalosporins, penicillins, and aztreonam. These *β*-lactamases are known as extended-spectrum *β*-lactamases (ESBLs) [23]. Recently, these enzymes have been found in other genera and species, including* Proteus mirabilis*,* Salmonella* spp., and* Enterobacter* spp. [24].

ESBLs-producing bacteria are a major problem in the management of certain bacterial infections and are a growing concern in those hospitals where antibiotics use is frequent and the patients are in critical conditions [7]. ESBL-producing bacterial pathogens limit the therapeutic options for the treatment; therefore, strategies for laboratory detection of ESBL-producing bacteria as well as antimicrobial susceptibility testing are important. However, despite the challenges involved with the technical sensitivity in the routine detection of ESBLs-producing bacteria, use of combined disk diffusion test is of great help in the resource limited rural setting like KRRH. This phenotypical technique based on disk diffusion is the cheapest strategy in the rapid detection of ESBLs to meet local demands in a resourced constrained setting. Therefore, in this current study, we detected presence of ESBL-producing bacteria using indicator antibiotics including cefpodoxime, ceftazidime, cefotaxime, ceftriaxone, and aztreonam, using special screening zone or MIC breakpoints which helped in recognition of ESBL production. The selection of indicator antibiotics was based on their likelihood of being readily hydrolyzed by one of the many types of ESBL enzymes produced by the mentioned bacteria.

In this study, prevalence of ESBLs-producing bacteria was found to be high (89 (89%)) compared to the prevalence recorded at Khartoum Teaching Hospital, Sudan (45.1%) [24], and in Lebanon (15.4%) [2], which are both developing countries.* K. pneumoniae* and* E. coli* are the most common species phenotypically producing ESBLs from clinical specimens and a similar observation has been reported around the world [1, 6, 7]. Alarmingly, most of these bacterial isolates are common culprits of nosocomial bacterial pathogens [2, 6, 23]. Significantly, they are the main cause of UTI and septicaemia, in agreement with observation seen by Yadav and Chauhan and Ogefere et al., respectively [7] [1]. The predominance of* ESBLs*-producing* Klebsiella *sp. (46 (52%)) reported here was also observed in the study by Jain et al. with the prevalence of 86.6% in India [25]. ESBLs-producing* E. coli* (39 (44%)) was the second most predominant bacterial isolate, which is in line with Shaikh et al.'s results in Australia [2] and Ibrahim et al.'s results in Sudan [24]. However, this disagrees with Jain et al. who reported that* Enterobacter *sp. were the second most prevalent ESBLs-producer in clinical isolates [25].

## 5. Conclusion

In a resourced constrained setting like KRRH, the use of phenotypical technique to identify and detect ESBL-producing bacteria based on combined disk diffusion is the cheapest strategy to routinely use to meet local demands compared to more expensive and unaffordable genotypic detection techniques.

The prevalence of ESBL-producing* E. coli*,* Klebsiella *sp., and* Proteus *sp. detected in this study is of great concern at both KRRH and other healthcare settings in Uganda, which requires sound and committed sustainable infection control measures including antimicrobial management and routine detection of ESBL-producing isolates.

## Figures and Tables

**Table 1:** Bacterial isolates from clinical specimens.

Bacterial isolates	Clinical specimen		
	Urine (*n* = 67)	Pus (*n* = 70)	CSF (*n* = 13)
*Escherichia coli*	29	13	2
*K. pneumoniae*	15	21	0
*K. oxytoca*	6	9	0
*P. mirabilis*	3	1	1
*n* (%)	53 (79%)	44 (63%)	3 (23%)

Key note: *n* = total number of isolates.

**Table 2:** Prevalence of phenotypic ESBL-producing isolates by combined disc diffusion test.

Combined antibiotic discs	Bacterial isolates (*n* = 100)				
	*K. oxytoca* (*n* = 15)	*K. pneumoniae* (*n* = 36)	*E. coli* (*n* = 44)	*P. mirabilis* (*n* = 5)	*n* (%)
Ceftazidime	10 (66.7%)	29 (80.6%)	36 (86.4%)	4 (80.0%)	79 (79%)
Cefotaxime	11 (73.3%)	35 (97.2%)	43 (97.7%)	5 (100%)	94 (94%)
Cefpodoxime	15 (100%)	33 (91.7%)	39 (88.6%)	4 (80.0%)	91 (91%)
Ceftriaxone	15 (100%)	35 (97.2%)	39 (100%)	2 (40%)	91 (91%)
Mean (*n* = 89)	13 (87%)	33 (92%)	39 (87%)	4 (80%)	89 (89%)
